# t-PA Suppresses the Immune Response and Aggravates Neurological Deficit in a Murine Model of Ischemic Stroke

**DOI:** 10.3389/fimmu.2019.00591

**Published:** 2019-03-27

**Authors:** Dominik F. Draxler, Felix Lee, Heidi Ho, Charithani B. Keragala, Robert L. Medcalf, Be'eri Niego

**Affiliations:** Molecular Neurotrauma and Haemostasis, Australian Centre for Blood Diseases, Monash University, Melbourne, VIC, Australia

**Keywords:** stroke, MCAo, t-PA, plasminogen, immune system, immunosuppression

## Abstract

**Introduction:** Acute ischemic stroke (AIS) is a potent trigger of immunosuppression, resulting in increased infection risk. While thrombolytic therapy with tissue-type plasminogen activator (t-PA) is still the only pharmacological treatment for AIS, plasmin, the effector protease, has been reported to suppress dendritic cells (DCs), known for their potent antigen-presenting capacity. Accordingly, in the major group of thrombolyzed AIS patients who fail to reanalyze (>60%), t-PA might trigger unintended and potentially harmful immunosuppressive consequences instead of beneficial reperfusion. To test this hypothesis, we performed an exploratory study to investigate the immunomodulatory properties of t-PA treatment in a mouse model of ischemic stroke.

**Methods:** C57Bl/6J wild-type mice and plasminogen-deficient (plg^−/−^) mice were subjected to middle cerebral artery occlusion (MCAo) for 60 min followed by mouse t-PA treatment (0.9 mg/kg) at reperfusion. Behavioral testing was performed 23 h after occlusion, pursued by determination of blood counts and plasma cytokines at 24 h. Spleens and cervical lymph nodes (cLN) were also harvested and characterized by flow cytometry.

**Results:** MCAo resulted in profound attenuation of immune activation, as anticipated. t-PA treatment not only worsened neurological deficit, but further reduced lymphocyte and monocyte counts in blood, enhanced plasma levels of both IL-10 and TNFα and decreased various conventional DC subsets in the spleen and cLN, consistent with enhanced immunosuppression and systemic inflammation after stroke. Many of these effects were abolished in plg^−/−^ mice, suggesting plasmin as a key mediator of t-PA-induced immunosuppression.

**Conclusion:** t-PA, via plasmin generation, may weaken the immune response post-stroke, potentially enhancing infection risk and impairing neurological recovery. Due to the large number of comparisons performed in this study, additional pre-clinical work is required to confirm these significant possibilities. Future studies will also need to ascertain the functional implications of t-PA-mediated immunosuppression for thrombolyzed AIS patients, particularly for those with failed recanalization.

## Introduction

Ischemic stroke continues to be a leading cause of disability and mortality worldwide (1). Thrombolytic therapy with tissue-type plasminogen activator (t-PA) remains the only approved pharmacological treatment for acute ischemic stroke (AIS) despite significant limitations, including a short therapeutic window (3–4.5 h after onset) and failed recanalization in ~60% of all treated patients (2, 3). In addition, t-PA treatment carries life-threatening risks, mainly for development of symptomatic intracerebral hemorrhage (sICH), which has been associated with t-PA-mediated disruption of the blood brain-barrier (BBB) (4). Together with additional evidence linking the thrombolytic to neurotoxicity (5, 6), t-PA complications are major reasons for concern when the decision to treat is made. These issues are particularly relevant for patients who fail to reanalyze in response to the treatment, hence failing to gain any therapeutic benefit of the drug.

Recent studies further suggest that t-PA, via plasmin, might additionally affect the immune response post-AIS. For example, the Preventive Antibiotics in Stroke Study (PASS) demonstrated that administration of preventive antibiotics improved outcome only in thrombolysed AIS patients, a result which could indicate a link between t-PA treatment and post-stroke infection (7). Plasmin has been well-characterized as a pro-inflammatory mediator (8), and more recently associated with immunosuppressive effects via the shutdown of dendritic cells (DC), thereby inducing a tolerogenic phenotype (9). Such immunomodulatory consequences of thrombolytic therapy would be undesirable for stroke patients, who are particularly susceptible to infections (such as pneumonia) due to the development of a central nervous system (CNS) injury-induced immunosuppression (10) and increased gut permeability, allowing the migration of bacteria from the gastrointestinal tract into the circulation (11).

Given the potential implications of t-PA and plasmin on the immune system, we conducted an exploratory study to examine whether t-PA thrombolysis can modulate the immune response post-stroke, promote inflammation and immunosuppression and negatively affect functional recovery.

## Materials and Methods

### Animals

Male C57BL/6J wild-type mice (*n* = 43) at 8–12 weeks of age weighing ~25 g were obtained from the Alfred Medical Research and Education Precinct (AMREP) Animal Services. Male and female plasminogen-deficient (plg^−/−^) mice (*n* = 33) were obtained from a heterozygote breeding colony maintained at the AMREP Animal Services. Animals were housed on a 12 h dark/light cycle at 21 ± 2°C with 40–70% relative humidity. Water and food were provided *ad libitum*. All animal procedures were undertaken in accordance with the National Health and Medical Research Council (NHMRC) “Code of Practice for the Care and Use of Animals for Experimental Purposes in Australia” and were approved by an Animal Ethics Committee of AMREP, Monash University.

### Middle Cerebral Artery Occlusion (MCAo) Model of Stroke

MCAo or sham surgeries were performed under aseptic conditions as previously described (12), with minor modifications. In brief, mice were anesthetized with isoflurane (5% induction then 2% maintenance in 100% O2). Rectal temperature was monitored and maintained at 37.0°C throughout the procedure. The fur around the neck was shaved and skin sterilized with 80% ethanol (v/v). A longitudinal incision was then made under the neck, followed by blunt dissection of neck muscles and the vagus nerve to expose the carotid bifurcation. Transient focal cerebral ischemia was induced by occlusion of the MCA for 60 min using a 6-0 silicone-coated monofilament (Doccol Corporation, MA, USA), inserted into the internal carotid artery via an external carotid stump. The mouse remained under anesthesia throughout the occlusion period. The filament was then retracted to allow reperfusion. Both successful occlusion (>70% reduction in cerebral blood flow; CBF) and reperfusion were verified by transcranial Laser-Doppler flowmetry (AD instruments, NSW, Australia). Head and neck wounds were stitched and treated with Xylocaine® and Betadine® (Sanofi, Australia) ointments and mice were transferred for recovery on a heat mat. Sham-operated mice were anesthetized for similar periods and the right carotid bifurcation exposed, without insertion of filament. Following drug administration (below), mice were hydrated with subcutaneous injection of sterile saline (1 ml), then housed individually in warmed cages for overnight recovery.

36 C57BL/6J and 29 plg^−/−^ mice underwent the successful experimental protocol (defined as uncomplicated surgery with successful occlusion, complete drug treatment and good recovery). Seven additional C57BL/6J mice and 4 plg^−/−^ mice (all MCAo surgeries) failed to meet these criteria and were not analyzed. No mortalities were recorded in the sham groups. the overall mortality from MCAo by 24 h after successful surgeries was 5.9% (1/17) in C57BL/6J mice [10% (1/10) for vehicle- and 0 (0/7) for t-PA-treated animals] and 31.25% (5/16) in plg^−/−^ mice [28.6% (2/7) for vehicle- and 33.3% (3/9) for t-PA-treated animals]. The higher mortality rates of plg^−/−^ compared to wild-type mice were expected due to increased cerebral microthrombosis in the plg deficient mice, resulting from the absence of plasmin-driven fibrinolysis (13).

### Drug Administration

Recombinant mouse t-PA (0.9 mg/kg; Molecular Innovations, MI, USA) or its vehicle (0.4M HEPES, pH 7.4) were administered immediately after reperfusion as a bolus injection via the tail vein (4 ml/kg; 100 μl per 25 g mouse). Mouse t-PA was used to avoid potential confounding effects due to possible immunogenicity of human recombinant t-PA (rt-PA), representing a foreign protein in the mouse. The clinical dose was selected for mouse t-PA (instead of the commonly used rt-PA dose of 10 mg/kg in rodents) since mouse t-PA, unlike rt-PA, has high affinity toward mouse plasminogen (14). To minimize bias, animals were randomly assigned to groups and treated by a separate person other than the surgeon.

### End Point Procedure

23 h post-MCAo mice underwent functional deficit assessment (below) and were then killed at 24 h by urethane (25% w/v, 12 ml/kg i.p.). 540 μl of blood was first collected from the vena cava into 60 μl of sodium citrate buffer (32 mg/ml sodium citrate dehydrate, 4.2 mg/ml citric acid monohydrate). The spleen was then removed, followed by transcardial perfusion with phosphate-buffered saline (20 ml). Cervical lymph nodes (cLN) were next collected and the brain was finally removed, divided into ipsilateral and contralateral hemispheres and processed as described below. All further analyses were performed by an operator blinded to group allocation of the animals.

### Behavioral Assessment

23 h after stroke mice were assessed for neurological deficit by summation of scores for forelimb flexion, torso twisting, lateral push resistance and general mobility, according to the method by Petullo et al. (15). Mice were then placed successively for 5 min each time in an open box (30 × 30 cm) and in a parallel rod floor apparatus (20 × 20 cm) of an ANY-maze (Stoelting, IL, USA), an automated behavior tracking system which records the spontaneous animal movements and extracts parameters such as distance, speed, latency to move, circling behavior, and foot faults. Finally, a hanging wire test for grip strength was performed as described (12), averaging 3 trials with 5 min rest intervals. Scores ranged from 0 for animals that fell immediately to a maximum of 180 s, typically achieved by sham-operated mice.

### Albumin ELISA

Detection of elevated albumin levels in the brain parenchyma after transcardial perfusion was used as an index of BBB breakdown as we previously described (12). To measure brain albumin, perfused brain hemispheres were homogenized to 300 mg/ml (wet weight) in PBS + 1% Triton X-100 (v/v) and snap-frozen on dry ice. Lysates were thawed before use and spun down at 16,100 × g for 10 min. Supernatants were diluted further in PBS, so the final sample concentration in the ELISA plate was 25 or 250 μg/ml (wet weight) for the ipsilateral or contralateral hemisphere, respectively. Brains of sham-operated mice were processed as the contralateral hemispheres. Albumin concentration was then quantified by ELISA using a commercial kit (Mouse Albumin ELISA Quantitation Set #E90-134; Bethyl Laboratories, TX, USA) according to the manufacturer's instructions. Total brain protein concentration was further determined by the Pierce® bicinchoninic acid (BCA) assay (ThermoFisher Scientific, Australia). Albumin content in the perfused brain was calculated according to the formula: Albumin (μg/mg total brain protein) = Albumin (μg/ml) / Total brain protein (mg/ml). The albumin levels obtained in the contralateral hemispheres were subtracted from the ipsilateral hemispheres in MCAo mice to account for perfusion efficiency.

### Plasma Cytokines

Plasma was isolated from citrated whole blood in sub-groups of sham and MCAo mice. A cytokine antibody array kit (Mouse ProcartaPlex™ Panel (ThermoFisher Scientific, Australia), detecting 8 different analytes (IL-6, IFN-γ, TNF-α, IL-β, IL-13, MCP-1, IL-10, and TGF-β) simultaneously on a Luminex platform, was performed by Crux Biolab (Melbourne, Australia) according to the manufacturer's instructions. Data was acquired on a Luminex platform MAGPIX. The median fluorescence intensity (MFI) was calculated for each analyte in samples and standards, and data was extracted using Procartal Plex Analyst software. Analyte concentrations were determined from the MFIs of the known standard concentrations.

### Blood Leukocyte Profiling

Differential white blood cell counts were assessed in sub-groups of sham and MCAo mice by analyzing citrated blood samples with the Hemavet 950FS analyser (Drew Scientific, FL, USA).

### Flow Cytometry

Flow cytometry analysis of cLN and spleen was performed in sub-groups of mice 24 h after MCAo or sham procedures in order to identify and characterize both myeloid and lymphoid cell populations, with a focus on DC and T cell subsets. Furthermore, neutrophils, macrophages, and monocytes as well as natural killer (NK) cells and B cells were assessed with our unique staining protocol.

After extraction, lymphatic organs were gently forced through a 40 μm cell strainer (BD Biosciences, Australia) to prepare a single cell suspension. Red blood cell (RBC) lysis was first performed on spleen samples with a RBC lysis buffer (BioLegend, Australia) according to the manufacturer's instructions. Cells were then centrifuged at 1,500 × g for 5 min and resuspended in PBS + 2% fetal calf serum (FCS). The single cell suspensions were stained with 2 distinct, fluorochrome-labeled antibody cocktails (summarized in Supplementary Table 1). The first cocktail identified the myeloid subsets and consisted of CD11c V450, MHC class II APCeFluor780, B220 AF700 (all from BD Biosciences), CD11b PECy7, Gr1 FITC, CD86 PE (all from eBiosciences), CD103 AF647, F4/80 PerCPCy5.5, CD8 BV650, and CD80 biotin (all from BioLegend) + streptavidin PE-CF594 (BD Biosciences). A second fluorochrome-labeled antibody cocktail, aimed at identifying and characterizing lymphoid subsets, consisted of CD3 APCeFluor780, CD69 V450, NK1.1 PE, CD19 FITC, (all from eBiosciences), CD8 BV650 (BioLegend), CD4 BV605 and B220 AF700 (both from BD Biosciences). All samples were further stained for live/dead cell discrimination with the LIVE/DEAD™ Fixable Aqua Dead Cell Stain Kit (Life Technologies, ThermoFisher Scientific, Australia). Cells were finally fixed with 1% paraformaldehyde (in PBS). Final resuspension also contained Truecount™ calibration beads (BD Biosciences) at a concentration of 100,000 beads/ml for exact enumeration of cells. Data were acquired on the LSR Fortessa flow cytometry analyzer (BD Biosciences) using the FACS DIVA software (BD Biosciences). Data analysis was performed with FlowJo software V10 (FlowJo, LLC, OR, USA). A diagram of the gating strategy as well as summary of the identified cell populations and functional markers used is provided in Supplementary Figure 1.

### Statistics

Statistical analysis was performed using GraphPad Prism 7 software. Outliers were determined in all data sets by Grubbs' test with α ≤ 0.05 and excluded from the analysis. No more than one outlier was identified in any group (see Figures). Where *n* numbers allowed and if data were not consistent of a group of zero values, data sets (excluding neurological scores) also passed the D'Agostino & Pearson normality test. Differences between two groups were determined by the Mann–Whitney *U*-test for neurological scores and by an unpaired, two-tailed Student's *t*-test elsewhere. Differences between four groups differing in two parameters (stroke vs. sham, t-PA vs. vehicle; excluding neurological scores) were analyzed by ordinary two-way ANOVA with Sidak's *post-hoc* analysis (with 2–6 comparisons per family). Probability values under 0.05 for *t*-tests or below the corresponding multiplicity adjusted *p*-values with family-wise significance level of 0.05 (depending on the number of comparisons made) for Sidak's *post-hoc* were considered significant.

## Results

### t-PA Exacerbates Motor Function Impairment After MCAo

**Figure 1:**
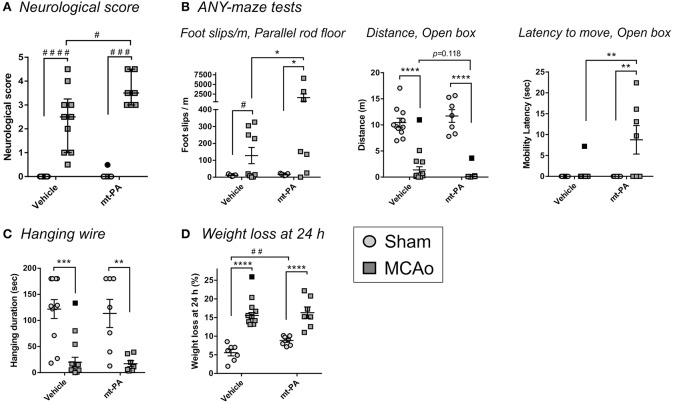
t-PA treatment worsens functional outcomes of middle cerebral artery occlusion (MCAo) in C57Bl/6 mice. Assessment of neurological deficit score **(A)**, motor capacity on ANY-maze **(B)**, hanging wire test **(C)**, and weight loss **(D)** 23 h after sham surgery or transient MCAo (1 h) followed by treatment at reperfusion with HEPES vehicle or mouse t-PA (mt-PA; 0.9 mg/kg). MCAo robustly affects all functional parameters compared to sham, as expected, but administration of mt-PA significantly worsens post-stroke performance compared to vehicle [**A,B** (Foot slips & Latency) and a trend in **B** (Distance)]. Hanging wire test is not further affected by mt-PA **(C)**. mt-PA-treated sham mice also significantly loose more weight compared to vehicle-treated controls **(D)**. Data is shown as individual animals with median + IQR **(A)** or mean ± SEM **(B–D)**. *n* = 7–11 for sham vehicle, 7 for sham t-PA, 8–10 for MCAo vehicle, 7 for MCAo t-PA. *p < 0.05, **p < 0.01, ***p < 0.001, ****p < 0.0001 by two-way ANOVA with Sidak's *post-hoc*, ^#^*p* < 0.05, ^##^*p* < 0.01, ^###^*p* < 0.001, ^####^*p* < 0.0001 by the Mann–Whitney *U*-test **(A)** or by the student *t*-test **(D)**. Outliers are denoted in black symbols and excluded from the analysis.

In wild-type mice, MCAo induced a profound impairment of motor function at 23 h, which was further aggravated in t-PA-treated mice as indicated by a significant increase in neurological score (*p* < 0.05; Figure 1A) and in foot slips (per distance) measured in the parallel rod floor apparatus of the ANY-maze system (*p* < 0.05; Figure 1B). t-PA did not shorten the total distance traveled post-stroke compared to vehicle (*p* = 0.118; Figure 1B), but significantly prolonged the latency to move (indicative of hesitation and stress) in MCAo mice (*p* < 0.01; Figure 1B) in the ANY-maze open box test. No additional differences in seven other mobility parameters assessed by the open box test were detected (mean speed, total time mobile, total time immobile, number of mobile episodes, number of immobile episodes, clockwise rotations, and anti-clockwise rotations). While the hanging wire test confirmed profound effects of MCAo, as expected, no differences were observed between treatment groups (Figure 1C). Interestingly, t-PA-treated sham mice showed a significantly greater weight loss compared to their vehicle-treated counterparts (*p* < 0.01), with no comparable effects in the MCAo groups (Figure 1D). Taken together, t-PA worsened the neurological deficit in our mechanical stroke model despite its early use (1 h post occlusion), suggesting that systemic events, rather than local, detrimental effects in the brain, were possibly occurring.

### Early t-PA Treatment Does Not Enhance BBB Disruption After Stroke

**Figure 2:**
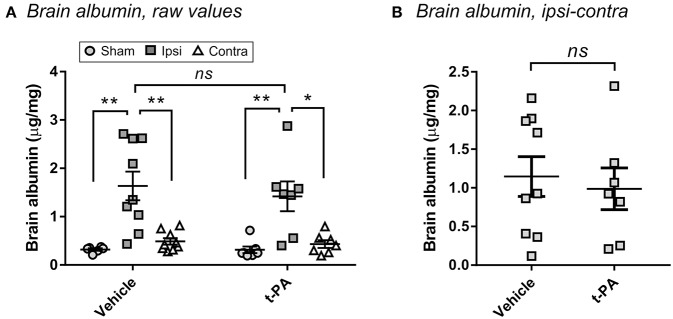
t-PA does not intensify blood-brain barrier (BBB) disruption when administered 1 h post-stroke. Albumin levels in the perfused brain 23 h after administration at reperfusion of HEPES vehicle or mouse t-PA (mt-PA; 0.9 mg/kg) to C57Bl/6 mice undergoing sham surgery or transient MCAo (1 h). Data is presented either as raw values (μg albumin per mg brain protein; **A**) or as corrected values (MCAo mice only after subtraction of the contralateral from the ipsilateral level to correct for perfusion efficiency; **B**). Stroke induces significant albumin accumulation in the injured hemisphere, indicative of BBB disruption, however no differences are observed between vehicle and mt-PA at this early time point. Data is shown as individual animals with mean ± SEM. *n* = 6 for sham vehicle, 7 for sham t-PA, 9 for MCAo vehicle, 7 for MCAo t-PA. *p < 0.05, **p < 0.01, *ns*, not significant by student *t*-test.

To confirm that t-PA was not directly affecting the brain in our model, we measured BBB disruption 24 h post-stroke—a hallmark of the undesirable effects of t-PA after experimental thrombolysis (12). As shown in Figure 2A, MCAo significantly enhanced albumin extravasation into the ipsilateral hemisphere compared to the contralateral hemisphere and to brains of sham-operated mice (*p* < 0.05), indicating BBB opening by the ischemic insult. Yet, no differences were observed between vehicle- and t-PA-treated animals at this early time of drug administration. A lack of t-PA effect also remained after subtraction of the contralateral albumin levels from the ipsilateral levels to account for perfusion efficiency (Figure 2B), cementing the conclusion that other factors rather than potentiation of brain injury by t-PA were playing a functional role in our study.

### t-PA Causes Additional Reduction in Circulating Lymphocytes and Monocytes After MCAo

Since plasmin and t-PA gained recent attention for their immunomodulatory capabilities, we next performed a series of evaluations to determine the additional impact of thrombolysis on the immune system during stroke.

**Figure 3:**
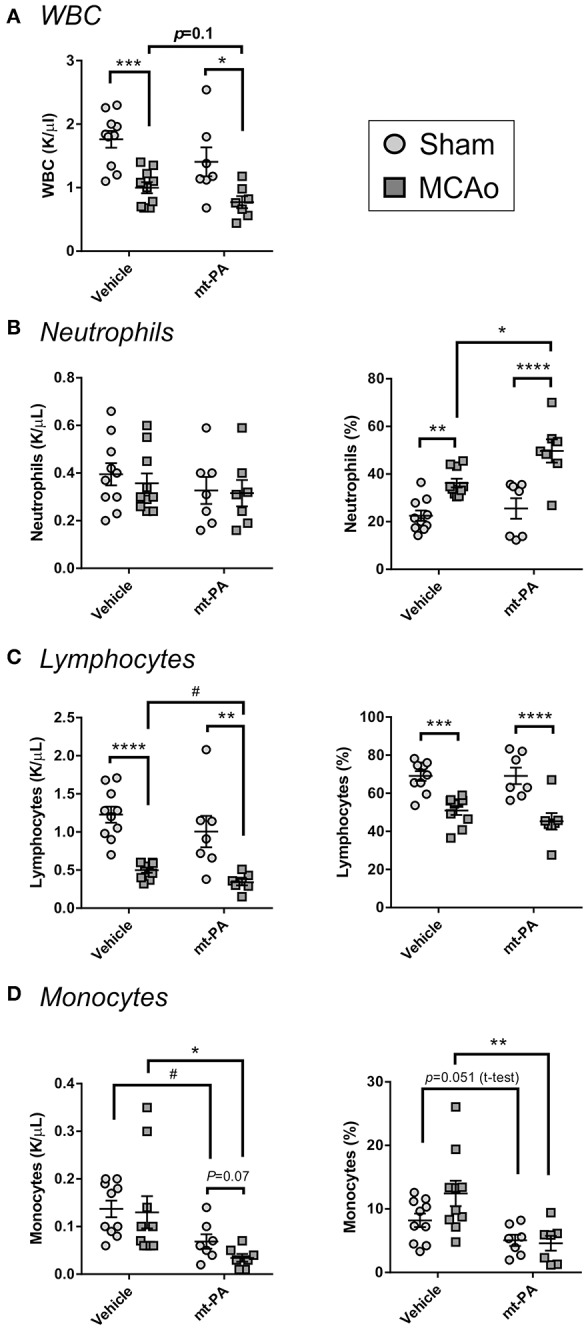
t-PA exacerbates stroke-triggered changes in blood leukocyte counts and proportions. Hemavet blood counts (left panels) and proportions (right panels) of total white blood cells (WBC; **A**), neutrophils **(B)**, lymphocytes **(C)**, and monocytes **(D)** 23 h after administration at reperfusion of HEPES vehicle or mouse t-PA (mt-PA; 0.9 mg/kg) to C57Bl/6 mice undergoing sham surgery or transient MCAo (1 h). Stroke causes robust leukopenia **(A)** and lypmhopenia **(C)**, which are exacerbated by treatment with t-PA. t-PA also causes significant monocytopenia in both sham and MCAo mice compared to vehicle **(D)**. Neutrophil proportion in blood is significantly elevated after stroke and further increases by t-PA, but neutrophil numbers remain unchanged **(B)**. These observations indicate potentiation of stroke effects on blood leukocytes by t-PA. Data is shown as individual animals with mean ± SEM. *n* = 10 for sham vehicle, 7 for sham mt-PA, 10 for MCAo vehicle, 7 for MCAo mt-PA. *p < 0.05, **p < 0.01, ***p < 0.001, ****p < 0.0001 by two-ANOVA with Sidak's *post-hoc*, ^#^*p* < 0.05 by the student *t*-test.

In line with the well-described immunosuppressive effects of stroke (10), MCAo induced a significant reduction of circulating white blood cells in both vehicle- and t-PA-treated groups, with a tendency for enhancement of this effect by t-PA (*p* = 0.1; Figure 3A). While absolute neutrophil numbers remained unaffected 24 h after MCAo (Figure 3B; left panel), lymphocyte levels and proportions in blood were not only markedly reduced in both treatment groups (*p* < 0.01; Figure 3C, left and right panels, respectively), but were further suppressed by t-PA compared to vehicle in animals subjected to MCAo (*p* < 0.05; Figure 3C, left panel). Moreover, monocyte levels were significantly decreased in t-PA-treated mice, both in the sham and the MCAo groups (*p* < 0.05; Figure 3D). These observations suggest that t-PA treatment might worsen stroke-induced leukopenia by exacarbation of lypmhopenia and monocytopenia, with major potenial consequences for infection resistance post-stroke.

### t-PA Alters Both Pro-inflammatory and Immunosuppressive Plasma Cytokines During Stroke

**Figure 4:**
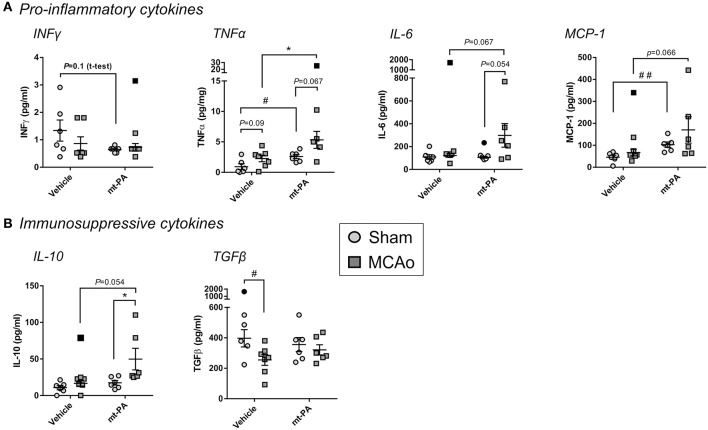
Plasma cytokine levels after stroke are affected by t-PA treatment. Pro-inflammatory **(A)** and immunosuppressive **(B)** cytokine levels measured by multiplex ELISA in mouse plasma 23 h after administration at reperfusion of HEPES vehicle or mouse t-PA (mt-PA; 0.9 mg/kg) to C57Bl/6 mice undergoing sham surgery or transient MCAo (1 h). t-PA treatment significantly elevates pro-inflammatory and immunosuppressive cytokines in shams as well as in MCAo mice compared to vehicle, most notably TNFα, MCP-1 **(A)** and IL-10 **(B)**, respectively. Trends for reduction of INFγ **(A)**, elevation of IL-6 **(A)** and reversal of stroke-induced TGFβ suppression **(B)** are further observed in t-PA-treated mice. This cytokine profile demonstrates pleotropic effects of t-PA on immune function, which at least in part could contribute to immunosuppression after stroke. Data is shown as individual animals with mean ± SEM. *n* = 6 for sham vehicle, 6 for sham mt-PA, 7 for MCAo vehicle, 6 for MCAo mt-PA. *p < 0.05 by two-ANOVA with Sidak's *post-hoc*, ^#^*p* < 0.05, ^##^*p* < 0.01 by the student *t*-test. Outliers are denoted in black symbols and excluded from the analysis.

To capture additional overview of the systemic immune response with or without t-PA in our stroke model, we next measured major pro-inflammatory and immunosuppressive plasma cytokines 24 h after MCAo. The baseline levels of IFNγ, the central T helper type 1 (Th1) cytokine, was lower in t-PA- compared to vehicle-treated sham animals, however this observation did not reach significance (*p* = 0.1; Figure 4A). Strikingly, t-PA also significantly enhanced the pro-inflammatory cytokine TNFα, both under baseline (sham) and MCAo conditions (*p* < 0.05; Figure 4A). Further, IL-6 concentrations showed a near-significant elevation only in t-PA-treated MCAo mice (*p* = 0.054–0.067; Figure 4A) while MCP-1 was significantly higher in t-PA-treated shams (*p* < 0.05), underlying its trending rise also after MCAo (*p* = 0.066; Figure 4A).

Interestingly, plasma concentrations of the immunosuppressive cytokine IL-10 were also significantly increased after MCAo in t-PA-treated (*p* < 0.05), but not in vehicle-treated mice (Figure 4B), whereas a significant reduction in TGFβ in the vehicle group (*p* < 0.05) was abolished with t-PA (Figure 4B). Taken together, t-PA treatment shifts both the pro-inflammatory and immunosuppressive cytokine profiles after stroke.

### t-PA Modulates the Cellular Immune Profiles of Cervical Lymph Nodes (cLN) and Spleen

**Figure 5:**
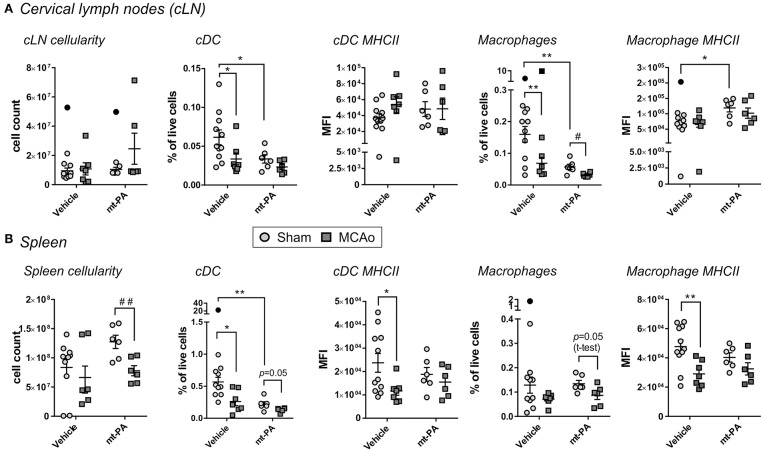
t-PA impacts on professional antigen-presenting cells (APCs) in lymphoid organs. Flow cytometry analysis of total cell count (cellularity; left panels), conventional dendritic cells (cDC) proportions (2nd left panels) and major histocompatibility complex class II (MHC II) expression (middle panels) as well as macrophage proportions (2nd right panels) and MHCII expression (right panels) in cervical lymph nodes **(**cLN; **A)** and spleen **(B)** 23 h after administration of HEPES vehicle or mouse t-PA (mt-PA; 0.9 mg/kg) to C57Bl/6 mice undergoing sham surgery or transient MCAo (1 h). In the cLN **(A)**, t-PA supresses cDC and macrophage numbers in sham-operated mice, but increases macrophage MHC II expression **(A)**. Similar, t-PA-mediated reduction in cDCs is observed in the spleen **(B)**, without effect on macrophage numbers **(B)**. Notably, significant stroke-induced reductions in MHC II expression seen in splenic cDCs and macrophages are lost with t-PA treatment, potentially due to overall suppression of MHC II also in sham-treated animals. Taken together, t-PA affects key APCs in lymphoid organs irrespective of stroke, an effect which could impact the immune response also after thrombolysis. Data is shown as individual animals with mean ± SEM. *n* = 10–11 for sham vehicle, 6 for sham mt-PA, 7 for MCAo vehicle, 6 for MCAo mt-PA. *p < 0.05, **p< 0.01 by two-ANOVA with Sidak's *post-hoc*, ^#^*p* < 0.05, ^##^*p* < 0.01 by student *t*-test. Outliers are denoted in black symbols and excluded from the analysis.

We next sought to investigate the influence of t-PA on systemic and local lymphatic organs by flowcytometric evaluation of spleen and cLN, respectively, 24 h post MCAo. Some changes in the cellular immune composition were induced by stroke following vehicle administration, including reductions in proportions of conventional dendritic cells (cDC) and macrophages in the cLN (*p* < 0.05; Figure 5A), decrease in splenic cDC proportion (*p* < 0.05; Figure 5B) and attenuation of MHCII expression on splenic cDC and macrophages (*p* < 0.05; Figure 5B). Notably, in addition to the changes induced by stroke alone, unique t-PA effects on the innate cellular immune profile could be observed; for example, while the overall cLN cellularity after MCAo was unaffected in any group (Figure 5A, left panel), cDC and macrophage proportions decreased in the lymph nodes of sham-operated mice after t-PA exposure (*p* < 0.05 and *p* < 0.01, respectively; Figure 5A). These results are indicative of a substantial effect of t-PA irrespective of the stroke-induced insult. The maturation marker MHCII remained unchanged on cLN cDC under any treatment (Figure 5A, middle panel), but was enhanced on macrophages from cLNs of shams following t-PA administration (*p* < 0.05; Figure 5A, right panel). Expression of the activation markers CD80 and CD86 after stroke remained unaffected by vehicle and t-PA treatment on cDC and macrophages from the cLN at the 24 h time point (data not shown). Additionally, no changes were observed in the frequency of cells and the expression level of activation markers in monocytes, CD4+ and CD8+ T cells, NK cells and B cells in the cLN at the 24 h time point (data not shown).

In the spleen, MCAo-induced reduction in cellularity was more pronounced and reached significance only in t-PA-treated animals (*p* < 0.01; Figure 5B, left panel). Splenic proportions of cDC were not only reduced after stroke in vehicle-treated mice (as mentioned earlier; *p* < 0.05), but were also strongly diminished after t-PA treatment in shams (i.e., under baseline conditions; *p* < 0.01; Figure 5B, 2nd panel from left), creating a 2-fold impact. MHCII expression on splenic cDCs was attenuated only in vehicle-treated (*p* < 0.05), but not in t-PA-treated animals after MCAo (Figure 5B, middle panel). Splenic macrophage proportions trended toward reduction in both treatment groups (Figure 5B, 2nd panel from right), however, macrophage expression of MHCII was profoundly downregulated after MCAo only in vehicle-treated (*p* < 0.01), yet not in t-PA-treated mice (Figure 5B, right panel). Similar to the cLN, no changes were detected in the expression intensity of CD80 and CD86 on splenic cDC and macrophages, as well as the frequency and activation status of monocytes, CD4+ and CD8+ T cells, NK cells and B cells at the 24 h time point (data not shown). Taken together, t-PA induces changes to both the local and systemic immune responses, with most effects already being triggered on the sham background, but some representing a specific immune modification following MCAo.

### t-PA-Induced Changes of the Immune Response During Stroke Include Plasmin-Dependent and—Independent Elements

**Figure 6:**
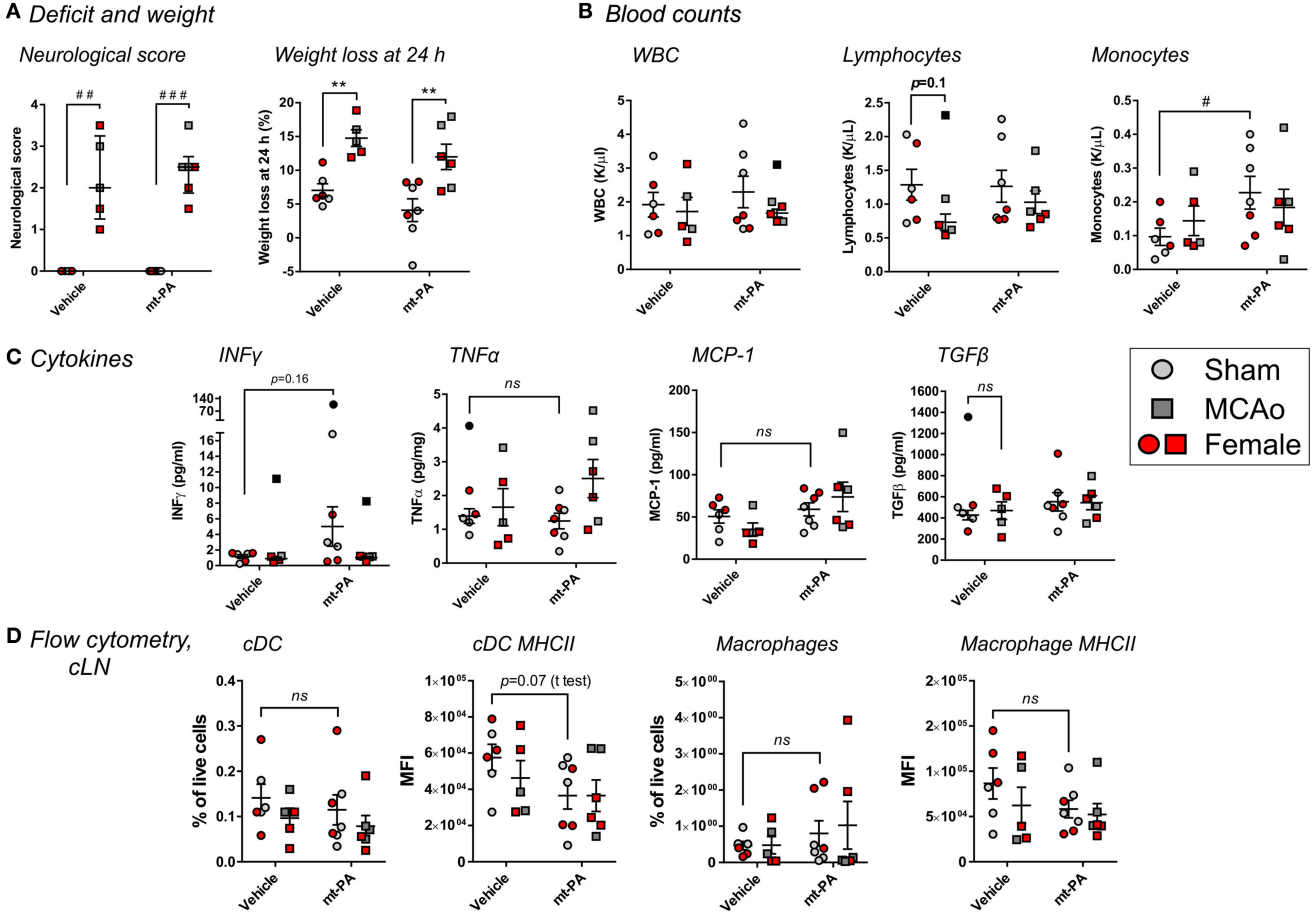
t-PA interactions with immune cells during stroke have a plasmin-dependent component. Effects of mouse t-PA (mt-PA; 0.9 mg/kg) or its HEPES vehicle on various functional and immunological parameters in plasminogen-knockout (plg^−/−^) mice 23 h after sham surgery or transient MCAo (1 h). **(A)** In plg^−/−^ mice, t-PA does not exacerbate neurological scores and weight loss, contrary to the observation in wild-type mice (Figure 1). **(B)** In contrast to blood profiles of C57Bl/6 mice after stroke (Figure 3), no t-PA potentiation of leukopenia and lypmhopenia, and monocytosis instead of monocytopenia, are observed in plg^−/−^ mice. **(C)** t-PA-induced changes of plasma cytokines seen in wild-type mice (Figure 4) are either reversed (INFγ ↑ instead of ↓) or diminish (no upregulation of TNFα and MCP-1, and no downregulation of TGFβ) in plg^−/−^ mice. **(D)** Finally, no t-PA-mediated reductions in cDC and macrophages and altered profiles of MHC II expression are observed in the cervical lymph nodes (cLN) of plg^−/−^ mice, in contrast to C57Bl/6 wild-type mice (Figure 5). Taken together, plasminogen mediates many of the t-PA effects on immune cells during stroke. Data is shown as individual animals (gray for male and red for female mice) with mean ± SEM. *n* = 6 for sham vehicle, 7 for sham mt-PA, 5 for MCAo vehicle, 6 for MCAo mt-PA. **p < 0.01 by two-ANOVA with Sidak's *post-hoc*, ^#^*p* < 0.05, ^##^*p* < 0.01, ^###^*p* < 0.0001 by the Mann–Whitney *U*-test **(A)** or by the student *t*-test **(B)**. Outliers are denoted in black symbols and excluded from the analysis.

To determine the involvement of plasmin in the observed immunomodulatory effects of t-PA after stroke, we performed similar experiments in plg^−/−^ mice, lacking the ability to generate plasmin after t-PA administration (16). Similar to wild-type mice, plg^−/−^ animals displayed profound increases in neurological scores (*p* < 0.001; Figure 6A) and weight loss (*p* < 0.01; Figure 6A) 24 h after MCAo. Yet, no t-PA exacerbation of deficits was observed, in contrast to wild-type mice. Strikingly and contrary to wild-type mice, no t-PA-mediated blood leukopenia (Figure 6B) and lymphopenia (Figure 6B) were observed in plg^−/−^ mice, whereas baseline (sham) levels of monocytes were even increased, rather than reduced, by t-PA treatment (*p* < 0.05; Figure 6B). Major alterations in cytokine responses compared to wild-type mice were seen on the plasminogen-deficient background, with no significant, t-PA-related changes after MCAo in IFNγ, TNFα, and MCP-1 or the immunosupressive cytokine TGFβ (Figure 6C). Finally, in contrast to wild-type mice, our flow cytometry analysis did not highlight significant changes in the proportion and MHCII expression of cDC (Figure 6D; two left panels, respectively) or macrophages (Figure 6D; two right panels, respectively) in the cLN of plg^−/−^ mice in response to vehicle or t-PA treatments post-stroke. Despite these dichotomies, other t-PA-related effects (or lack of effects) described in wild-type mice on neurological deficits, blood counts, cytokines, and lymphoid organs were similarly observed in plg^−/−^ mice, for example splenic cDC proportions were significantly reduced by t-PA in both mouse strains (Figure 5B; data not shown in plg^−/−^ mice). Overall, this investigation indicates a co-existence of both plasmin-dependent and -independent immune-modulatory effects of t-PA.

## Discussion

To date, t-PA is the only approved pharmacological treatment for AIS (17). While there is no doubt about its therapeutic advantage in patients in whom recanalization is achieved, the majority of patients who receive t-PA do not recanalize (2, 3), hence do not benefit from the treatment. Since t-PA and plasmin are also broadly involved in processes independent of fibrinolysis (4, 18), there is a need to unravel possible non-haemostatic effects of t-PA when it is used at the pharmacological, “exaggerated” concentrations. Such an understanding will enable optimization of therapy to reduce thrombolysis-associated risks for AIS patients.

MCAo for 1 h induced a profound motor function deficit 23 h after occlusion, but this outcome was significantly worsened in C57Bl/6J mice receiving t-PA, demonstrating the potential of t-PA to also mediate harmful effects during its use in stroke. This worsening in motor function may be explained, at least in part, by the previously described neurotoxic effects of t-PA (5, 6), which might be of particular relevance when such high pharmacological doses of the protease are administered. While the possible contribution of t-PA-mediated neurotoxicity and local brain inflammation to outcome has not been directly explored in this study, it is unlikely to play a role due to the early time point after stroke onset chosen for t-PA administration. More specifically, a large number of thrombolysis studies in rodent stroke models demonstrate that t-PA is not neurotoxic and does not affect infarct size when administered early after stroke (including within 1 h as performed here and even when the dose of human t-PA is increased to 10 mg/kg) (4). Hence, it is likely that no substantial, t-PA-induced intracranial changes (neurotoxicity, inflammation) would have been observed in our study 24 h post-stroke using 0.9 mg/kg t-PA.

We further considered the effects of t-PA on BBB permeability, potentially contributing to brain oedema and intracranial hemorrhage (19, 20), as an explanation for the pronounced neurological deterioration post-t-PA in wild-type mice. t-PA-mediated disruption of the BBB via activation of the Rho-kinase pathway has been suggested as an underlying mechanism for the ability of thrombolysis to promote sICH (19, 20). However, this effect could be ruled out by assessing albumin exatravasation into the brain 24 h post-stroke, which did not differ between vehicle and t-PA treated animals. The lack of exacerbation of BBB disruption by t-PA (Figure 2) further implies that only negligible amounts of t-PA will actually reach the brain parenchyma at this early time point of administation, strengthening our hypothesis that additional processes might have been taking place.

Another possibility to explain the observed t-PA-induced impairment in motor function is the interaction of the plasminogen-activating system with the immune response (8). The MCAo model of stroke has been previously demonstrated to induce profound immunosuppression (11, 21), allowing us to study the modulation of this effect by t-PA.

Indeed, immunosuppressive effects of stroke were further aggravated by t-PA, such as the profound decrease in circulating lymphocytes, a hallmark feature of CNS injury-induced immunosuppression (10). Additionally, t-PA potentiated the reduction in circulating monocytes, and enhanced the levels of the pro-inflammatory cytokines TNFα, IL-6, MCP-1, and the immunosuppressive cytokine IL-10. The proportions of cDC and macrophages in the cLN (the draining lymph node system of the brain) (22) and of cDC in the spleen were also profoundly reduced by t-PA already in sham animals. Both macrophages and cDC are potent antigen-presenting cells, linking the innate and adaptive immune responses (10, 23). In combination, these results clearly indicate the contribution of t-PA to both pro-inflammatory and immunosuppressive changes in C57Bl/6J wild-type mice.

The Coexistence of harmful systemic inflammation and immunosupression has previously been described in critically ill patients (24). However, our paradigm that thrombolytic therapy might enhance both processes during stroke should not be taken for granted, since in the context of post-stroke infection, systemic inflammation is thought to subside at 24–48 h and subsequently lead to a more immunosuppressive state (25, 26). Thus, our observation of a simultaneous rise in both pro-inflammatory and immunosuppresive cytokines (e.g., TNF-α and IL-10, respectively) would not typically be seen (27). One important factor that needs to be considered is the effect of t-PA and plasmin on the background of stroke. Stroke itself can be predominantly immunosuppressive 24 h post-onset. However, plasmin is well-documented to induce pleotropic effects on the immune system, both pro-inflammatory (8) and immunosuppressive (9). The net effect of stroke and thrombolysis might therefore differ and become more complex than the effect of stroke alone, potentially underlying the observations made here.

With this in mind, both local and systemic inflammation as well as immunosuppression with increased susceptibility to infection (10) provide a possible explanation for the worsening effect in motor function observed after t-PA treatment in our study. Intriguingly, this idea is also in line with the observations made in the PASS trial, in which only thrombolysed AIS patients benefited from preventive antibiotics (7).

An important statistical issue, which might affect our conclusions thus far, arises from the large number of comparisons performed in this study. Since we did not control the family-wise error rate (FWER) for the hypothesis-testing of multiple outcome variables, there is a higher probability of detecting significant results purely due to chance, creating type I errors (“false positives”) (a problem also known as the “multiple testing problem”). However, the study is exploratory in nature and should mainly be used to inform the design of adequately powered confirmatory trials for specific outcome variables, so tight control of FWER is not of paramount importance. In addition, corrective procedures for multiple comparisons can sometimes be overly conservative and in practice introduce type II errors (i.e., “false negatives”), thereby restricting the predictive capacity of exploratory work like the one presented here. Taken together, these statistical caveats, while imperative, do not take from the significance of our findings.

We repeated these experiments in plg^−/−^ mice to assess whether t-PA itself, or its enzymatic product plasmin, is responsible for the observed changes. Interestingly, while many changes were entirely absent in plg^−/−^ mice, indicating a plasmin-dependent process (e.g., lymphopenia, the increase in pro-inflammatory plasma cytokines, as well as the proportional alterations of cDC and macrophages in the cLN), others were present in plg^−/−^ mice to a comparable extent as wild-type mice, suggesting a truly t-PA-mediated, plasmin-independent activity as the underlying mechanism (for example splenic cDC proportions). For a few parameters, t-PA administration in fact induced opposite effects in C57Bl/6J and plg^−/−^ mice (e.g., circulating monocyte levels). Notably, plasmin-independent immune-modulatory effects of t-PA have been previously reported for microglial function in the brain (28, 29), supporting our observations.

A few caveats should be mentioned also in the mechanistic study with plg^−/−^ mice. First, the C57Bl/6J wild-type mice were all males, whereas both genders were used in the plg^−/−^ experiments. While similar numbers of male and female plg^−/−^ mice were included in all treatment groups, absolute numbers per gender remained low. Yet, within the limited cohorts investigated, we have not observed any relevant differences between genders in any of the parameters tested and the data do not support a gender-related bias of our observations. Additonally, based on power calculations from our initial exploratory study in wild-type C57Bl/6J mice, it seems that while observations in sham-operated plg^−/−^ mice were sufficiently powered, some observations in plg^−/−^ mice undergoing MCAo were underpowered (including the lack of deterioration in neurological deficit and absence of monocytopenia following t-PA administration). Future studies aiming to unravel the full complexity of interactions between t-PA and the immune system could benefit from larger cohort sizes; nevertheless, clear trends are apparent across most variables tested suggesting that plg^−/−^ mice substantially differ from wild-type mice in their response to t-PA. In addition, several parameters were significantly different between sham and MCAo groups in vehicle-treated wild-type mice, but not in vehicle-treated plg^−/−^ mice (e.g., leukocyte and lymphocyte blood counts (Figures 3A,C vs. Figure 6B) and proportions of cDC and macrophages in the cLN (Figure 5A vs. Figure 6D). This further supports our conclusion that the plasminogen-activating system is involved in immune modulation during thrombolysis.

Taken together, both plasmin-dependent and -independent immune-modulatory effects of t-PA are triggered during thrombolysis, contributing to immunosuppression and inflammation, and potentially worsening motor function outcome. While we have not provided a causative link between t-PA-mediated immune alterations and neurological deterioration, a connection appears plausible and should be examined in subsequent studies. Another important area for future exploration is the long-term effects of thrombolysis on the immune system, in the weeks to months following stroke. Nevertheless, most post-stroke infections occur within the acute phase of stroke (up to 7 days from onset) (30, 31). Hence, it is likely that most t-PA-mediated functional immune changes will occur robustly within the earlier period post-stroke and that our findings represent some of the core effects of t-PA and plasmin in this context.

It is worth highlighting that our results might in fact be an underestimation due to a few experimental limitations: (i) First, we chose a relatively short occlusion time (60 min) in the suture model, which not only mimics the eligible time-period for clinical thrombolysis, but also ensures reperfusion. More pronounced immunosuppresive effects of t-PA on the immune system may have occurred in situations where the ischaemic period is prolonged, or where reperfusion is not achieved at all. Unfortunately, prolonged transient (>1 h) or permanent occlusions were less suitable for our study since their outcomes are closer in the mouse to a malignant human stroke, where devastating outcomes develop with or without t-PA (32). (ii) In addition, we chose to administer t-PA as a bolus, rather than an infusion, to minimize prolonged exposure to anesthesia, which we found to suppress the immune system irrespective of treatment (not shown). Nonetheless, in such a bolus regimen a rapid quenching of t-PA and shorter exposure of immune cells to plasmin occur, limiting the immune-modulatory effects of these proteases. The significant interactions of t-PA and plasmin with the immune system seen in this study, despite the limitations mentioned, emphasize the potency of the phenomena described.

In any case, detrimental effects of t-PA/plasmin have to be taken into account when a decision for thrombolysis is made. Treatment adjustment for patients who are unlikely to recanalize might have to be considered, raising the need for a point of care test to predict treatment outcome. While thrombectomy is becoming more and more available as an alternative treatment option, intravenous t-PA administration is still indicated in this procedure as a bridging therapy (33). Treatment refinement and a more personalized therapeutic approach may be beneficial for AIS patients to better account for the unwanted side effects of t-PA and to achieve maximal treatment success and safety.

## Data Availability

All datasets generated for this study are included in the manuscript and the Supplementary Files.

## Author Contributions

DD, RM, and BN: project concept and writing of the manuscript; DD, FL, HH, and BN: experimentation; DD, FL, HH, CK, and BN: data analysis and statistical analysis; DD, FL, HH, CK, RM, and BN: editing of manuscript.

### Conflict of Interest Statement

The authors declare that the research was conducted in the absence of any commercial or financial relationships that could be construed as a potential conflict of interest.
