# Supplementary material for: t-PA Suppresses the Immune Response and Aggravates Neurological Deficit in a Murine Model of Ischemic Stroke
Source: Front Immunol. 2019 Mar 27;10:591. doi: 10.3389/fimmu.2019.00591 (PMC6445967; doi:10.3389/fimmu.2019.00591)
Supplement: Supplementary file 1 [file Data_Sheet_1.docx]

Supplementary material to Draxler et al:
“t-PA suppresses the immune response and aggravates neurological deficit in a murine model of ischemic stroke”

”

**Supplementary table 1. Antibody panels used for flow cytometry**

| **antibody cocktail** | **fluorochrome-labelled antibody** | **supplier** |
| --- | --- | --- |
|  | CD11c V450, MHC class II APCeFluor780, B220 AF700 | BD Biosciences, USA |
| **myeloid subsets** | CD103 AF647, F4/80 PerCPCy5.5, CD8 BV650 and CD80 biotin | BioLegend, USA |
|  | CD11b PECy7, Gr1 FITC, CD86 PE | eBiosciences, USA |
|  | LIVE/DEAD™ Fixable Aqua Dead Cell Stain Kit | Life Technologies, USA |
|  | CD4 BV605, B220 AF700 | BD Biosciences, USA |
|  | CD8 BV650 | BioLegend, USA |
| **lymphoid subsets** | CD3 APCeFluor780, CD69 V450, NK1.1 PE, CD19 FITC | eBiosciences, USA |
|  | LIVE/DEAD™ Fixable Aqua Dead Cell Stain Kit | Life Technologies, USA |

**Supplementary Figure 1. Gating strategy used to identify leukocyte phenotypes and functional markers**

A summary table of leukocyte markers used for gating **(A)** and representative flow cytometer panels exemplifying the gating strategy for identification of myeloid cell subsets **(B)** and lymphoid cell subsets **(C)**. The panels shown in **(B)** and **(C)** are from a cervical lymph nodes (cLN) sample of a sham mouse. Spleen samples were gated equivalently.

**(A) A summary table of leukocyte markers used for gating**

| **Myeloid cell phenotypes** | | | | | | | | | |
| --- | --- | --- | --- | --- | --- | --- | --- | --- | --- |
| **cDC** | SSC low | B220- | F4/80- | MHCII+ | CD11c+ | | CD11b- | |  |
| **monocytes** | SSC med |  | F4/80+ | MHCII- | CD11c- | | CD11b+ | | Gr-1- |
| **macrophages** | SSC med |  | F4/80+ | MHCII+ |  | | CD11b+ | | Gr-1- |
|  | | | | | | | | | |
| **Myeloid cells – functional markers** | | | | | | | | | |
| **activation/maturation** | CD80 | | CD86 | | | | MHCII | | |
| **viability** | Live/Dead stain | | | | | | | | |
|  | | | | | | | | | |
| **Lymphoid cell phenotypes** | | | | | | | | | |
| **CD4+ T cells** | SSC low | B220- | CD3+ | CD4+ | CD8- |  | |  | |
| **CD8+ T cells** | SSC low | B220- | CD3+ | CD4- | CD8+ |  | |  | |
| **B cells** | SSC low | B220+ | CD3- | CD19+ |  |  | |  | |
| **NK cells** | SSC low | B220- | CD3+ | NK1.1+ |  |  | |  | |
|  | | | | | | | | | |
| **Lymphoid cells – functional markers** | | | | | | | | | |
| **activation** | CD69 | | | | | | | | |
| **viability** | Live/Dead stain | | | | | | | | |

**(B) Gating strategy used for flowcytometric characterization of myeloid cell subsets in cLN and spleen samples**
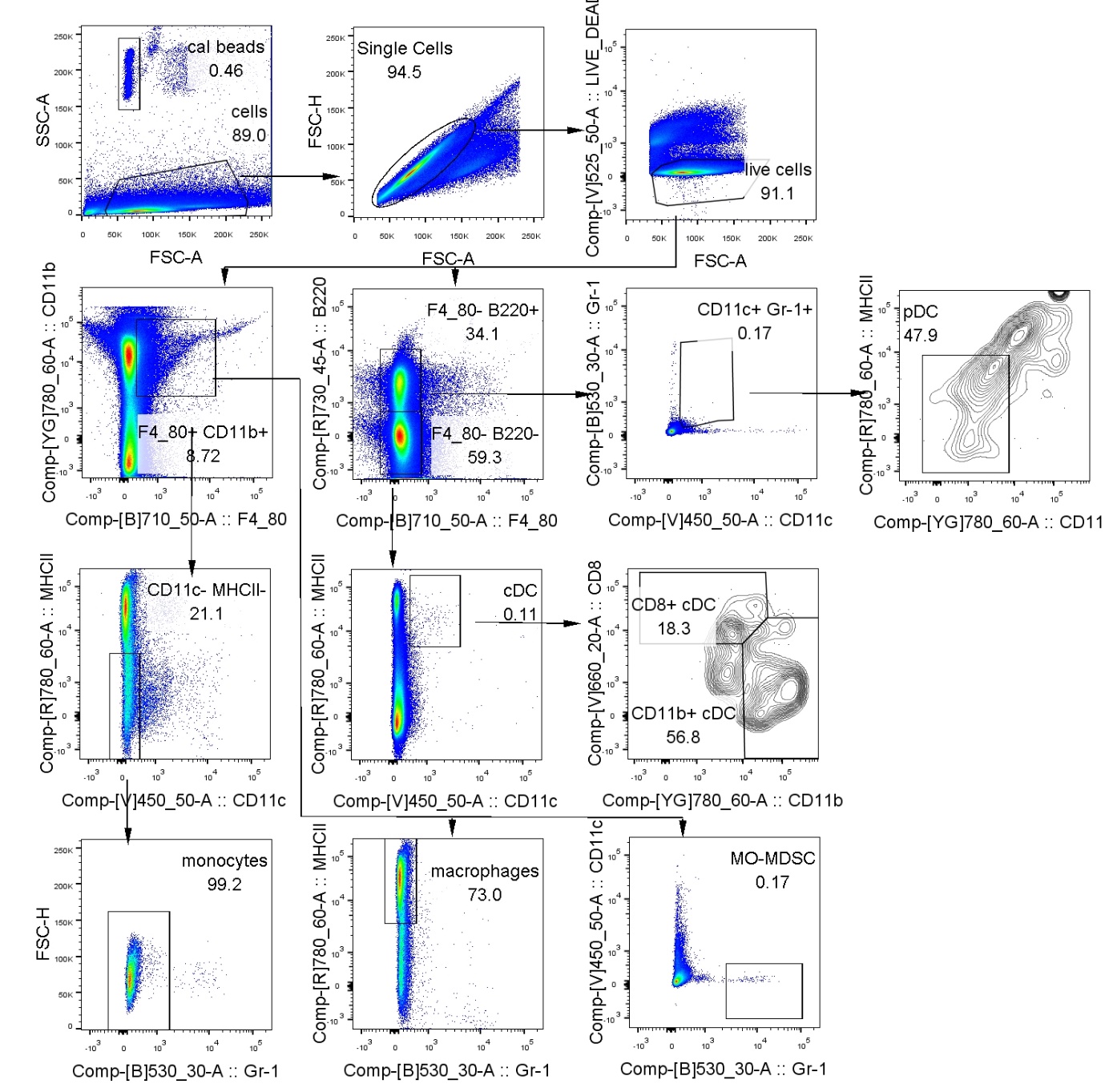
**.**

**
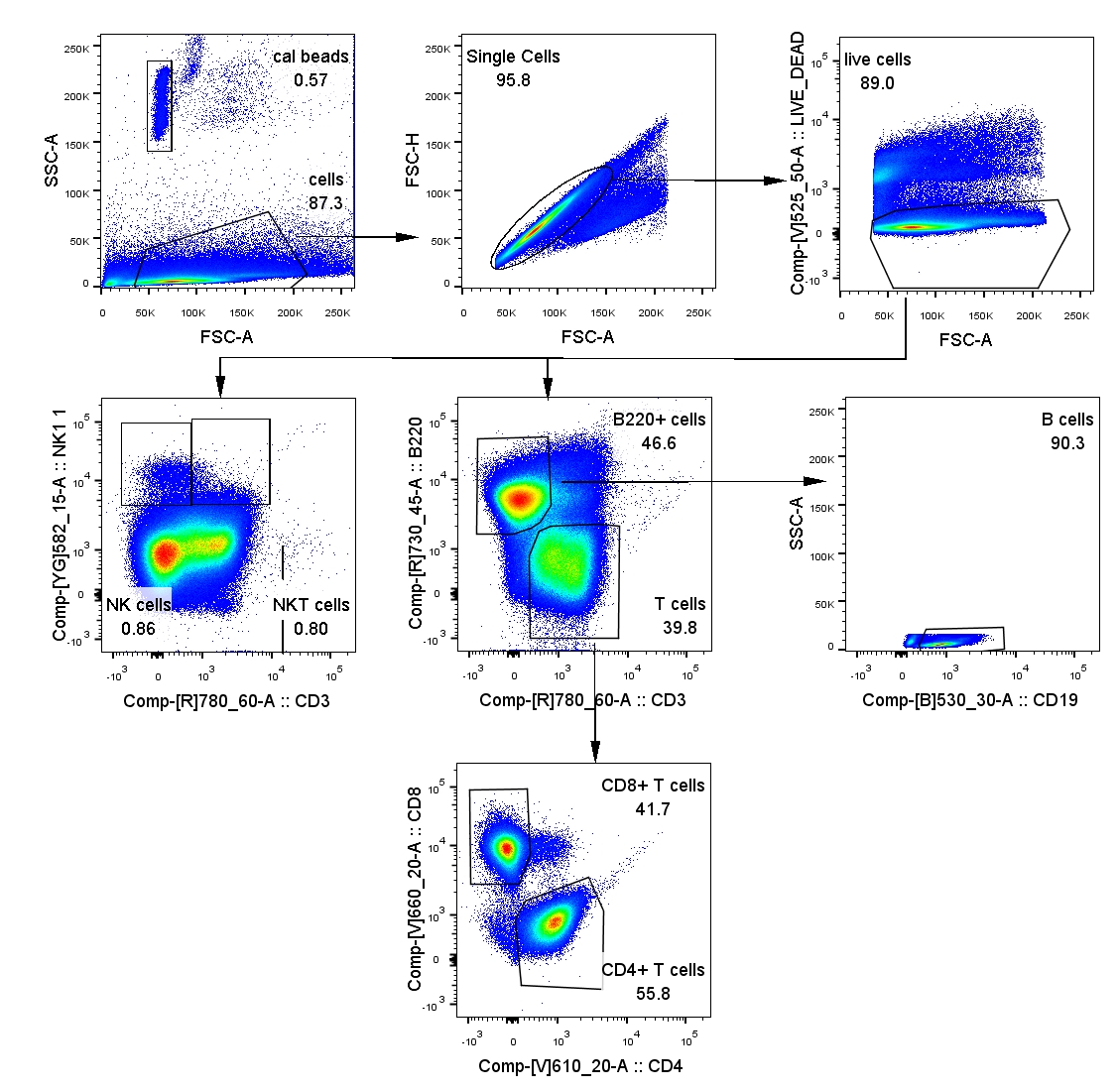
(C) Gating strategy used for flowcytometric characterization of lymphoid cell subsets in cLN and spleen samples.**
